# Expression of concern: A well-defined S-g-C_3_N_4_/Cu–NiS heterojunction interface towards enhanced spatial charge separation with excellent photocatalytic ability: synergetic effect, kinetics, antibacterial activity, and mechanism insights

**DOI:** 10.1039/d4ra90108h

**Published:** 2024-09-27

**Authors:** Haya A. Abubshait‡, Shahid Iqbal‡, Samar A. Abubshait, Mohammed T. Alotaibi, Norah Alwadai, Nada Alfryyan, Hashem O. Alsaab, Nasser S. Awwad, Hala A. Ibrahium

**Affiliations:** a Basic Sciences Department, Deanship of Preparatory Year and Supporting Studies, Imam Abdulrahman Bin Faisal University P. O. Box 1982 Dammam 31441 Saudi Arabia; b Department of Chemistry, School of Natural Sciences (SNS), National University of Science and Technology (NUST) H-12 Islamabad Pakistan shahidiqbal.chem@sns.nust.edu.pk; c Department of Chemistry, College of Science, Imam Abdulrahman Bin Faisal University P. O. Box 1982 Dammam 31441 Saudi Arabia; d Department of Chemistry, Turabah University College, Taif University P. O. Box 11099 Taif 21944 Saudi Arabia; e Department of Physics, College of Sciences, Princess Nourah Bint Abdulrahman University P. O. Box 84428 Riyadh 11671 Saudi Arabia; f Department of Pharmaceutics and Pharmaceutical Technology, Taif University P. O. Box 11099 Taif 21944 Saudi Arabia; g Chemistry Department, Faculty of Science, King Khalid University P. O. Box 9004 Abha 61413 Saudi Arabia; h Biology Department, Faculty of Science, King Khalid University P. O. Box 9004 Abha 61413 Saudi Arabia; i Department of Semi Pilot Plant, Nuclear Materials Authority P. O. Box 530 El Maadi Egypt

## Abstract

Expression of concern for ‘A well-defined S-g-C_3_N_4_/Cu–NiS heterojunction interface towards enhanced spatial charge separation with excellent photocatalytic ability: synergetic effect, kinetics, antibacterial activity, and mechanism insights’ by Haya A. Abubshait *et al.*, *RSC Adv.*, 2022, **12**, 3274–3286, https://doi.org/10.1039/D1RA07974C.

*RSC Advances* is publishing this expression of concern in order to alert readers that concerns have been raised over the integrity of the data published in this article. The authors have been contacted but have not provided the requested raw data. An expression of concern will continue to be associated with the article until a conclusive outcome is reached.

Laura Fisher

17th September 2024

Executive Editor, *RSC Advances*

